# Proteomic characterization of microdissected breast tissue environment provides a protein‐level overview of malignant transformation

**DOI:** 10.1002/pmic.201600213

**Published:** 2017-03-07

**Authors:** René B. H. Braakman, Christoph Stingl, Madeleine M. A. Tilanus‐Linthorst, Carolien H. M. van Deurzen, Mieke A. M. Timmermans, Marcel Smid, John A. Foekens, Theo M. Luider, John W. M. Martens, Arzu Umar

**Affiliations:** ^1^ Department of Medical OncologyErasmus MC Cancer InstituteErasmus University Medical CenterRotterdamthe Netherlands; ^2^ Postgraduate School of Molecular MedicineErasmus University Medical CenterRotterdamthe Netherlands; ^3^ Department of NeurologyErasmus University Medical CenterRotterdamthe Netherlands; ^4^ Department of SurgeryErasmus University Medical CenterRotterdamthe Netherlands; ^5^ Department of PathologyErasmus University Medical CenterRotterdamthe Netherlands

**Keywords:** Breast cancer, Cellular origin, Laser capture microdissection, Malignant transformation, Tissue environment

## Abstract

Both healthy and cancerous breast tissue is heterogeneous, which is a bottleneck for proteomics‐based biomarker analysis, as it obscures the cellular origin of a measured protein. We therefore aimed at obtaining a protein‐level interpretation of malignant transformation through global proteome analysis of a variety of laser capture microdissected cells originating from benign and malignant breast tissues. We compared proteomic differences between these tissues, both from cells of epithelial origin and the stromal environment, and performed string analysis. Differences in protein abundances corresponded with several hallmarks of cancer, including loss of cell adhesion, transformation to a migratory phenotype, and enhanced energy metabolism. Furthermore, despite enriching for (tumor) epithelial cells, many changes to the extracellular matrix were detected in microdissected cells of epithelial origin. The stromal compartment was heterogeneous and richer in the number of fibroblast and immune cells in malignant sections, compared to benign tissue sections. Furthermore, stroma could be clearly divided into reactive and nonreactive based on extracellular matrix disassembly proteins. We conclude that proteomics analysis of both microdissected epithelium and stroma gives an additional layer of information and more detailed insight into malignant transformation.

**Abbreviations**
AGC: target of automatic gain control
ECM: extracellular matrix
GOBP: gene ontology biological process
GOCC: gene ontology cellular compartiment
GOMF: gene ontology molecular function
HER2: human epidermal growth factor receptor 2
LCM: laser capture microdissection
LFQ: label‐free quantification
PR: progesterone receptor
UV: ultraviolet

## 1 Introduction

Breast cancer is a heterogeneous disease, which can be classified into a variety of different subtypes based on genetic and histopathological features 1, 2. Different subtypes are associated with different prognosis and outcome, and therefore detailed molecular characterization is important to obtain a better understanding of the disease, and to identify novel putative targets for therapy 3. Although molecular subtypes were first described by gene expression profiling, advancement in proteomics technology now also enables molecular subtyping of breast cancer at the protein level 4. However, identifying novel putative markers is still a challenging task, since tumor tissue is not only genetically heterogeneous, but also histologically. Breast tumor tissue consists of malignant tumor epithelium, and varying amounts of connective tissue and other cell types, including leukocytes, endothelial cells, fibroblasts, and normal epithelium. All these compartments contribute in concert to the development of the tumor. To molecularly characterize a tumor, detailed information of the total make‐up of the tumor is ideally obtained in a spatially resolved or cell type specific manner. Instead, when whole tissues or sections are used for protein profiling, a combined protein quantity of each region of tissue is obtained, which may complicate accurate characterization of the tumor, for example when proteins are present in distinct regions and have different functions. Additionally, in comparative analysis, for example, between tumors with different clinical outcome, variability in relative amounts of tumor epithelial cells and other cell types will add noise, and prevent detection of more subtle changes in protein expression. Strict inclusion criteria, for example, selection based on the number of tumor nuclei or tumor area, can alleviate possible confounding due to intratissue heterogeneity 5. However, this will limit the number of tissues that can be included in the analysis, and may also introduce bias into the analysis, for example, toward higher‐grade tumors.

**Significance of the study**

In this work, we describe intra‐ and extracellular proteomic changes in microdissected breast tissue sections with varying stages of disease, in a variety of cell types. Stroma in (malignant) breast tissue is heterogeneous, as evidenced by a clear distinction of two clusters of reactive and nonreactive stroma. This difference could be mainly attributed to extracellular matrix disassembly proteins. Even though extracellular proteomic changes were readily captured in microdissected epithelium, proteomic analysis of stroma provided more detailed analysis of malignant transformation. Thus, our study emphasizes the power of proteomics workflows that make use of laser capture microdissection samples from different cellular origin.

Stromal interference can be minimized by enriching tumor epithelial cells with laser capture microdissection (LCM). We have previously successfully implemented an LCM‐based proteomic workflow to develop both prognostic and predictive proteomic signatures in breast cancer based on epithelial tumor cells 6, 7. LCM‐based proteomics has also previously been used to characterize specific regions of interest, in both healthy and malignant tissues, such as blood vesicles 8 and amyloid plagues 9. Microdissected tissue characterization does not need to be limited to a single cell type, and various regions of tissues can be separately microdissected and analyzed, which allows for region‐specific proteomic maps to be obtained. Such region‐ and cell‐type‐specific proteomic maps could potentially give more depth to the characterization of disease stages, subtypes, and the identification of putative protein biomarkers.

As a proof of principle, we performed MS‐based proteomic analyses on microdissected normal, benign, premalignant, and malignant epithelium, as well as stroma and infiltrate from fresh‐frozen breast tissues with varying stages of disease. We characterized differences between normal and malignant epithelium and stroma, thereby showing the additional value of a cell‐type‐specific LCM‐proteomics workflow.

## 2 Materials and methods

### 2.1 Patients and tumor tissue

Snap frozen breast tumor tissues (*n* = 38) were used from our liquid N_2_ bio bank, which were selected based on invasive tumor cell percentage (nucleus count). This study was approved by the Medical Ethics Committee of the Erasmus MC Rotterdam, the Netherlands (MEC 02.953), and was performed in accordance to the Code of Conduct of the Federation of Medical Scientific Societies in the Netherlands (http://www.federa.org/). An overview of histological and molecular characteristics of selected tumors is provided in Supporting Information Table 1.

### 2.2 Cryosectioning and laser capture microdissection

Cryosectioning and LCM were performed as described previously 10. Briefly, 8 μm tissue sections were cut and melted on an ultraviolet (UV)‐treated polyethylene naphthalate slide (Carl Zeiss MicroImaging, Munich, Germany). Sections were fixed in ice‐cold 70% v/v ethanol/Milli‐Q water, dehydrated in 100% ethanol and stored at –80°C. Prior to LCM, sections were rehydrated in tap water, stained with hematoxylin, and dehydrated in increasing concentrations of ethanol in Milli‐Q water. LCM was performed using a type P‐MB device (Carl Zeiss MicroImaging) and completed within 1.5 h after staining. From each slide, an area of ∼500 000 μm^2^ was collected in opaque adhesive caps (Carl Zeiss MicroImaging), equivalent to ∼4000 epithelial cells (assuming 10 μm^3^ per cell 11). Collected cells were dissolved in 20 μL of 0.1% w/v RapiGest in 50 mM ammonium bicarbonate and stored at –80°C until further processing.

### 2.3 Protein digestion

Cells were lysed by sonication in a cup horn sonicating (water) bath, using an Ultrasonic Disruptor Sonifier II (Model W‐250/W‐450, Bransons Utrasonics, Danbury, CT, USA) for 1 min at 70% amplitude. Proteins were denatured at 99°C for 5 min, reduced by incubating with 5 mM DTT at 60°C for 30 min, and alkylated with 15 mM IAM in the dark at room temperature for 30 min. Subsequently, 1 μg trypsin was added (approximately 1:50 ratio protease:protein) and samples were incubated at 37°C for 4 h. After digestion, RapiGest SF was degraded by acidifying to 0.5% v/v TFA and incubating for 30 min at 37°C.

### 2.4 LC‐MS

Measurements were performed with a nano‐LC system (Ultimate 3000, Dionex, Amsterdam, The Netherlands) coupled online to a hybrid linear ion trap/Orbitrap mass spectrometer (LTQ‐Orbitrap‐XL, Thermo Fisher Scientific, San Jose, CA, USA) as previously reported 12. Samples were desalted on a trap column (PepMap C18, 300 μm id × 5 mm length, 5 μm particle size (d.p.), 100 Å pore size; Thermo Fisher Scientific) and then separated on the analytical column (PepMap C18, 75 μm i.d. × 500 mm, 3 μm d.p. and 100 Å pore size), with the following binary gradient: 0 to 25% solvent B in 120 min and 25 to 50% solvent B in a further 60 min, at a flow rate of 250 nL/min. Solvent A consisted of 2% ACN and 0.1% formic acid in HPLC water, and solvent B consisted of 80% ACN and 0.08% formic acid in HPLC water. For MS detection, a data‐dependent acquisition method was used. A high‐resolution survey scan from 400 to 1800 Th. was detected in the Orbitrap (target of automatic gain control = 10^6^, maximum injection time = 1000 ms, resolution = 30 000 at 400 Th). Based on this full scan, the five most intensive ions were consecutively isolated (target of automatic gain control = 10^4^ ions, maximum injection time = 400 ms), fragmented by collision induced dissociation (35% normalized collision energy), and detected in the ion trap. Selected precursor masses ±5 ppm were excluded for MS/MS fragmentation for 3 min or until the precursor intensity fell below an *S*/*N* of 1.5 for more than ten scans. The MS proteomics data have been deposited to the ProteomeXchange Consortium 13 via the PRIDE partner repository with the dataset identifier PXD003632.

### 2.5 Data analysis

Label‐free quantification was performed in MaxQuant 14 (v. 1.4.1.2). Data were searched using the Andromeda 15 search engine against a concatenated target‐decoy database (UniProt‐Swiss‐Prot 2014‐4 *Homo sapiens* canonical reference proteome). The cleavage rule was set to trypsin (no P‐rule), and an initial precursor mass window of 20 ppm and fragment mass tolerance of 0.5 Da was used. Oxidation of methionine and protein N‐terminal acetylation were set as variable modifications, and carbamidomethylation of cysteine as a fixed modification. Peptide and protein identifications were filtered to 1% FDR based on decoy counting. Alignment of peptides, for which fragmentation data were not obtained in each individual run, was enabled through the “match between run” option and the minimum number of peptides per protein required for quantitation was set to 2 (label‐free quantification minimum ratio count).

Protein annotation, PCA, and testing for differences (two‐sided Welch test) were performed in Perseus 16. Protein abundances were log transformed and replicates were averaged prior to testing. Annotation enrichment analysis was performed in Perseus or DAVID 17. A STRING 18 protein interaction network selected stromal proteins was created in Cytoscape 3.4.0 19 via Stringapp (http://www.cgl.ucsf.edu/cytoscape/stringApp/index.shtml). A minimum interaction score of 0.4 was applied. Hierarchical cluster analysis was performed in the program Cluster 20 using correlation as a distance metric and centroid linkage as agglomeration method. Resulting data were visualized in JAVA Treeview 21. Venn diagrams were plotted in the Venn diagram tool (http://bioinformatics.psb.ugent.be/webtools/Venn/). All other graphs were prepared in Microsoft Excel or GraphPad Prism 5.

## 3 Results

### 3.1 Protein identifications in microdissected regions

**Figure 1:**
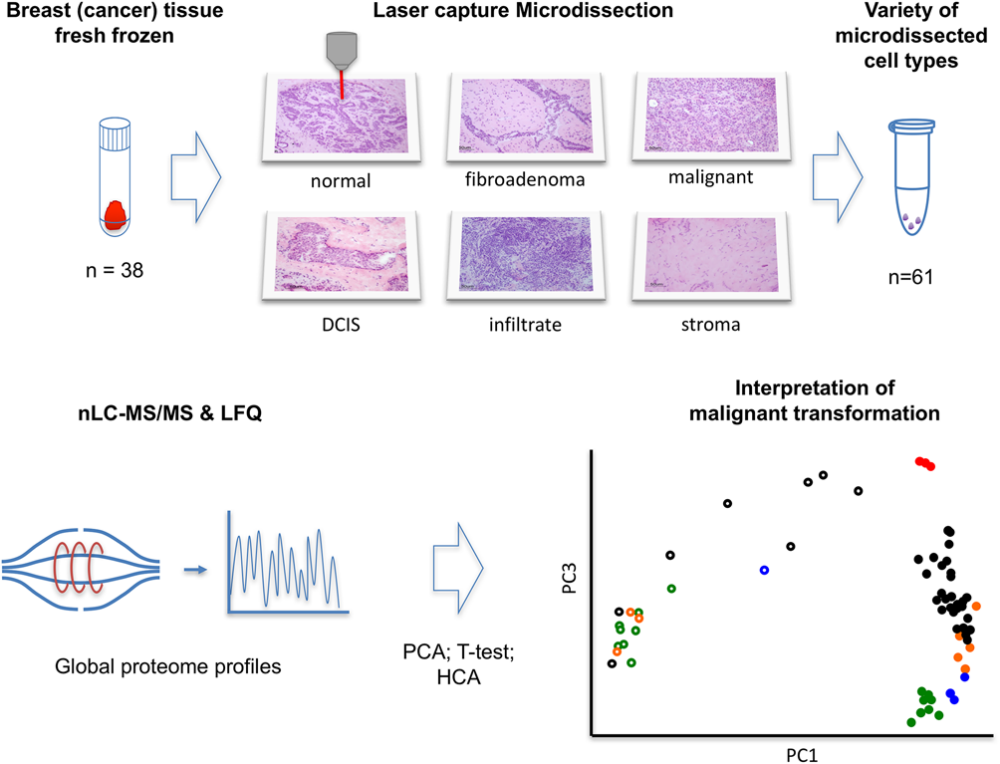
Schematic representation of the LCM‐proteomics workflow. Fresh frozen breast tissues were subjected to LCM, from which both epithelial and stromal cell regions were collected. Proteins were extracted, trypsin digested, and subjected to nano‐LC‐MS/MS analysis, after which the protein abundance data were analyzed by PCA.

We microdissected a variety of cell types (*n* = 61) from breast tissues (*n* = 38) for downstream global proteomic analysis (Fig. 1). Microdissected tissues included histologically normal epithelium and stroma from benign and malignant lesions (grouped as benign epithelium/stroma), benign epithelium and stroma from fibroadenomas, premalignant epithelial cells from ductal carcinoma in situ, and invasive tumor epithelium, stroma, and infiltrating leukocytes from breast cancer tissues (Fig. 1, Table 1, Supporting Information Table 1, representative HE staining shown in Supporting Information Fig. 1).

**Table 1:** Number of patients and microdissected areas from tissue cryosections

Patient lesion	*n* patients	Microdissected area	*n* microdissected
Benign	2	Benign epithelium	2
		Stroma	2
Fibroadenoma	2	Benign epithelium	2
		Stroma	1
CIS	3	Benign epithelium	2
		Carcinoma In‐Situ	1
		Stroma	2
Malignant	31	Benign epithelium	4
		Carcinoma In‐Situ	3
		Malignant epithelium	26
		Infiltrate	3
		Stroma	13
Total	38		61
			

In the complete tumor set (*n* = 38), 2995 protein groups were identified (excluding common contaminants), of which 719 were detected with fragmentation based evidence in all dissected areas (Supporting Information Fig. 2). Malignant tumors typically harbored larger groups of invasive cells that could be microdissected from their environment with relatively high purity, as assessed by histologically reviewing pictures taken from all microdissected areas. In samples from which microdissected tumor cells were collected (*n* = 26), 2663 protein groups were identified with at least one supportive MS/MS spectrum, of which 357 exclusively in the tumor group. Among these proteins was the epidermal growth factor receptor, a tumor marker that promotes tumor growth and survival, which is often overexpressed in tumors that do not express ER, progesterone receptor (PR), or human epidermal growth factor receptor (HER2) 22, clinically used markers on which therapy is based. We detected epidermal growth factor receptor with MS/MS fragmentation data in a microdissected triple negative tumor section, with 29 unique peptides, corresponding to 33% sequence coverage and an estimated abundance that was among the top ten most abundant proteins detected in malignant cells. In the group of microdissected ductal carcinoma in situ cells (*n* = 4), 1927 protein groups were detected, of which 1865 protein groups overlapped with malignant cells and 43 were unique to this group (Supporting Information Fig. 2 and Supporting Information Table 2).

Histologically normal cells that form mammary glands are smaller than tumor cells, and consist of two layers of epithelial cells. These cells were therefore more difficult to collect with high purity due to their smaller size. Despite the lower purity and relatively small sample of tissues from which benign epithelial cells were collected, 1850 protein groups were identified with MS/MS evidence, of which 43 were unique (Supporting Information Fig. 2 and Supporting Information Table 2). Among the proteins that were only detected with MS/MS evidence in microdissected histologically normal cells were several basement membrane proteins that are moderately expressed in breast tissues, such as nestin and tensin‐1.

Stroma, the connective tissue of the breast, is heterogeneous and consists of varying amounts of different cells, such as fibroblasts, endothelial cells, adipocytes, leukocytes and, in malignant tissue, tumor cells. We identified 1131 protein groups in malignant stroma and 418 protein groups in benign stroma, of which 83 stromal proteins were not detected in other microdissected cell types. This included proteins as CD163, a marker for macrophages, and Von Willebrandfactor, a marker for endothelial damage/dysfunction. Lastly, leukocytes were microdissected from malignant tissues that had high numbers of infiltrating cells. In the microdissected areas enriched with leukocytes, 1486 protein groups were identified, of which 82 were not identified in other microdissected regions (Supporting Information Fig. 2).

### 3.2 Dynamic range and detection of (clinically relevant) breast cancer markers

**Figure 2:**
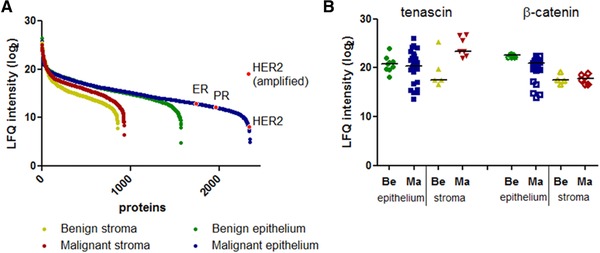
Dynamic range and abundance of selected proteins in microdissected areas. (A) Proteins from each microdissected region were sorted on median abundance. Selected tumor (HER2, ER, and PR) markers are highlighted. (B) Abundance of the stromal marker tenascin and epithelial marker β‐catenin, in malignant and histologically normal sections.

The dynamic range of peptide abundances at the peptide‐spectrum match level was approximately three to four orders of magnitude, which translated into an estimated protein dynamic range of six to seven orders of magnitude (Fig. 2A and Supporting Information Fig. 3). Intensity distributions varied between microdissected regions, as a result of a varying number of proteins with large differences in abundances. To verify data normalization, we selected two proteins, the epithelial marker β‐catenin and the stromal marker tenascin‐1 that covered most of the abundance range, and for which differences in expression between normal and malignant tissues are well described 23. While both markers were detected in all microdissected areas, abundance of β‐catenin was lower in malignant epithelium (*p* < 0.01) but not in stroma (*p* = 1), whereas tenascin had a higher abundance in malignant stroma (*p* = 0.03) but not epithelium (*p* = 0.7, Fig. 2B). Additionally, we compared levels of the ER, PR, and HER2. All of these markers were detected in the lower end of the abundance range in malignant epithelium in ER and HER2 positive tumors (Fig. 2A). Neither ER nor PR was detected in ER‐negative tumors. HER2 is amplified in approximately 20% of breast cancer cases, which leads to elevated transcript and protein levels. Median abundance of HER2 was among the lowest‐abundant proteins. However, samples with an HER2 amplification had HER2 protein levels that were over three orders of magnitude higher (Fig. 2A).

### 3.3 Protein expression and function in microdissected regions of interest

**Figure 3:**
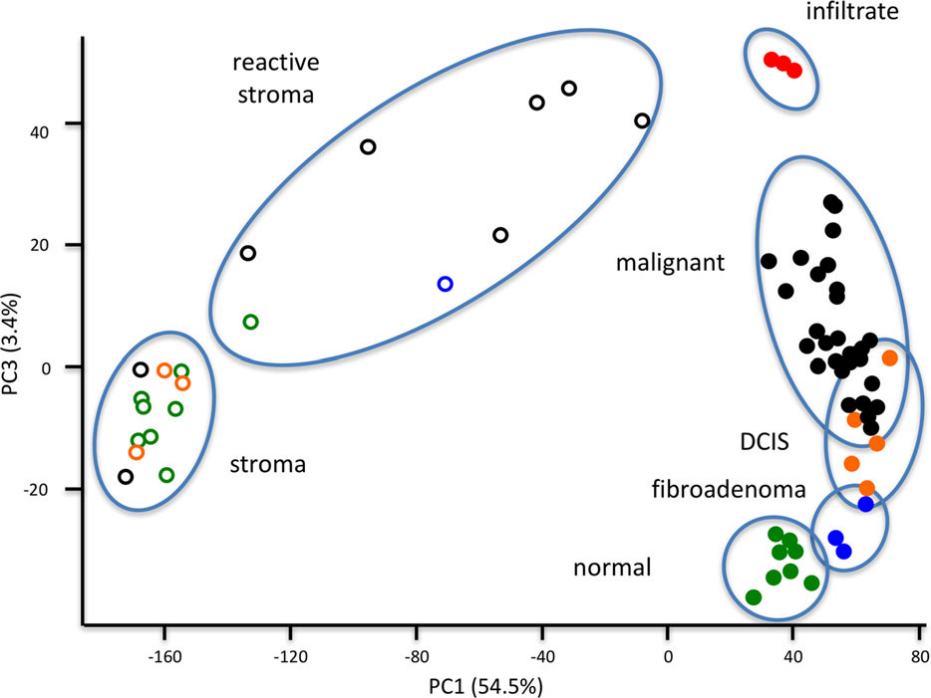
PCA scores plot of principal components 1 and 3. Microdissected samples clustered according to their histology, with principal component 1 discriminating between stroma and epithelium and principal component 3 discriminating between malignancy, on the basis of expression of immune regulatory proteins. Red filled squares: microdissected infiltrate from malignant tumor sections; black filled circles: microdissected malignant tumor epithelium; orange filled circles: microdissected ductal carcinoma in situ epithelium; blue filled circles: microdissected epithelial cells from fibroadenoma sections; green filled circles: microdissected epithelial cells from histologically normal sections. Black circles: stroma dissected from histologically malignant sections; blue circles: stroma dissected from a fibroadenoma section; orange circles: stroma dissected next to ductal carcinoma in situ lesions; green circles: stroma dissected from histologically normal sections.

**Figure 4:**
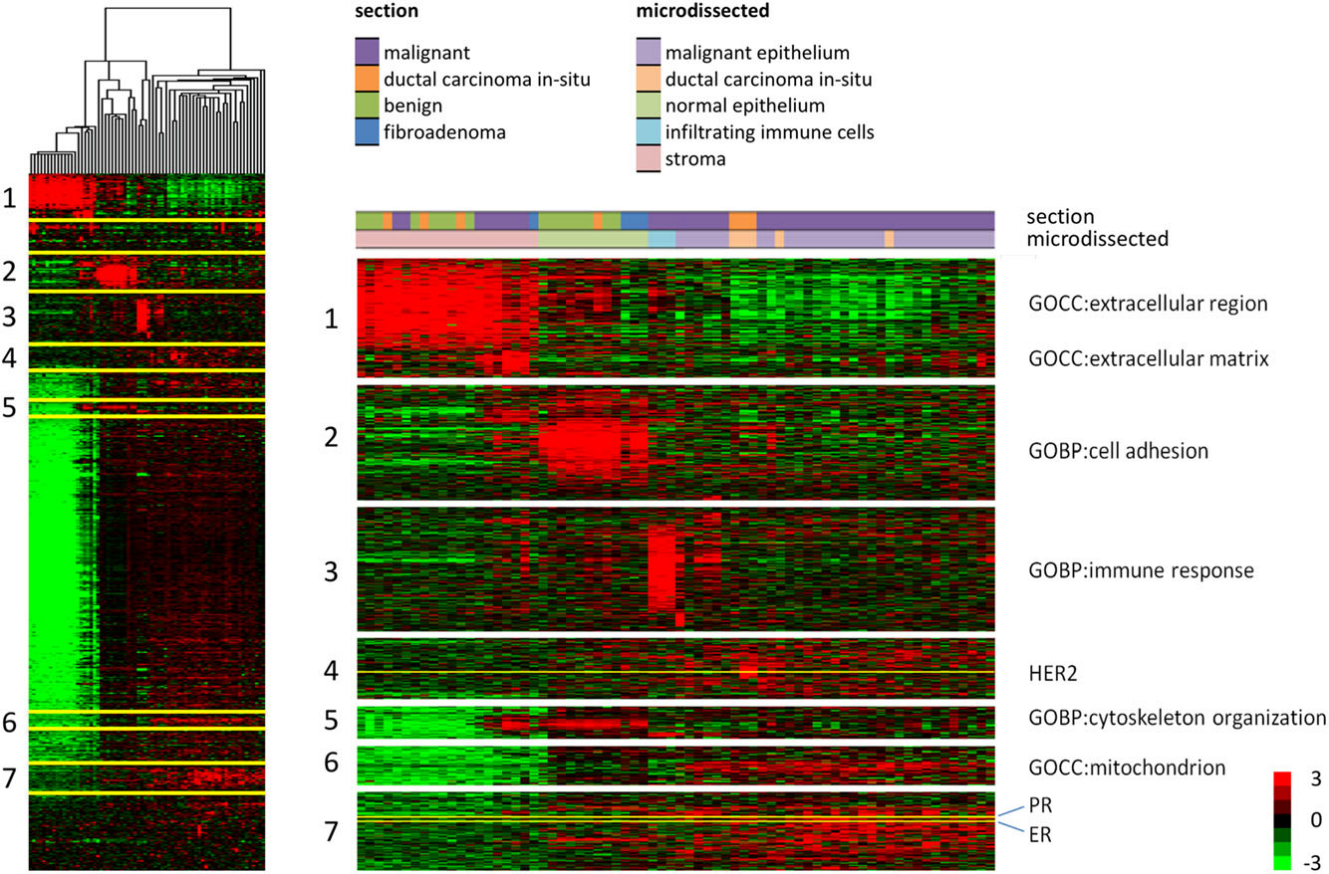
Heatmap of a cluster analysis based on protein abundances in each microdissected sample. Full cluster analysis and zoomed selected clusters that were strongly enriched in indicated cellular location or biological processes, as well as clusters around the markers ER, PR, and HER2.

We performed a PCA on protein abundances to classify microdissected regions of interest based on the largest differences between samples (Fig. 3). Principal components 1 and 3 most accurately clustered samples according to dissected areas and cellular types. The largest variance was observed in stroma, where two apparent clusters were formed, one homogeneous, more tightly clustered group consisting mainly of stroma dissected from histologically normal tissue sections (“stroma” cluster). A second heterogeneous stromal cluster explained over 50% of the variance in the dataset that consisted mainly of malignant stroma, as well as stroma dissected from a fibroadenoma (“reactive stroma” cluster, Fig. 3). Proteins with the highest loadings in principal component 1 were enriched for intracellular (GO cellular compartment: intracellular part, *p* < 0.01) and extracellular proteins (GO cellular compartment: extracellular region, *p* < 0.01) for negative and positive loadings, respectively (Figs. 3 and 4). In principal component 3, proteins with the highest loadings were enriched for immune regulatory proteins (GO biological process: immune system process, *p* < 0.01). Principal component 3 progressively separated epithelial cells of increasing malignancy, with the lowest scores for histologically normal epithelium, followed by epithelium from fibroadenomas, ductal carcinoma in situ and invasive carcinoma. Similarly, samples in stromal cluster 2, which had higher scores in principal component 3, were mainly of invasive origin.

The PCA showed that each microdissected cell type or compartment could nearly completely be distinguished based on expression of extracellular and immune regulatory proteins. To characterize these differences in more detail, we next performed a cluster analysis on the data and annotated the most abundant protein clusters (Fig. 4 and Supporting Information Table 3). Corresponding to the PCA, stromal samples clustered separately from epithelial cells and leukocytes, and the majority of normal and benign microdissected stromal samples formed a homogeneous cluster. Stromal samples from histologically malignant sections, along with stroma dissected from a fibroadenoma, were separated from this cluster and had a higher abundance of extracellular matrix (ECM), cell adhesion, and cytoskeletal proteins (Fig. 4, protein clusters 1, 2, and 5). Histologically normal epithelium and epithelium dissected from fibroadenomas were also separated from each other based on these protein clusters, where epithelium from fibroadenomas had a lower expression of the extracellular region and matrix proteins (protein cluster 1). Malignant cells were characterized with loss of cell adhesion and cytoskeletal organizing proteins and higher expression of mitochondrial proteins (protein clusters 2, 5, and 6). The clinically relevant markers ER, PR, and HER2 were detected in clusters 4 and 7. Individual protein abundances of ER, PR, and HER2 did not fully agree with immunohistochemical assessment of the tumor, with notable discrepancies found for ER in tumors that were immunohistochemically scored with negative PR expression, a marker for an active ER (Supporting Information Fig. 4). In tumors that were immunohistochemically ER positive, the majority of samples had detectable ER expression (0/6 ER negative vs. 16/21 ER positive, Fisher's exact *p* = 0.07).

### 3.4 Differences in protein expression between benign and malignant regions of interest

Proteins that were differentially abundant between malignant and normal epithelium could be associated with several hallmarks of cancer (Table 2, Supporting Information Tables 4 and 5). The GOs “ECM” (18% of differentially abundant proteins) and “basement membrane” (9%) together accounted for the majority of proteins that had a lower abundance in malignant epithelium. Additionally, proteins involved in cell adhesion (22% of differentially abundant proteins), response to wounding (12%), and cytoskeletal organization (11%) had a lower abundance. Among these proteins were ECM proteins, such as laminin B2 (fold‐change = –9.5, *p* < 0.01), serpin C1 (fold‐change = –7.4, *p* < 0.01), and collagen VII type A1 (fold‐change = –10.5, *p* < 0.01), but also intracellular proteins such as annexin A1 (fold‐change = –4.0, *p* < 0.01) and calponin (fold‐change = –8.6, *p* < 0.01; Supporting Information Table 4).

**Table 2:** Enriched GO terms in differentially abundant proteins between benign and malignant cells of epithelial origin

Term	Description	Count (%)	Fold enrichment	Benjamini–Hochberg adjusted *p*‐value	Direction
GO:0031012	ECM	44 (18%)	5.0	3.3 × 10^–19^	Down in malignant
GO:0007155	Cell adhesion	53 (22%)	3.9	3.4 × 10^–16^	Down in malignant
GO:0005604	Basement membrane	22 (9%)	7.3	6.8 × 10^–13^	Down in malignant
GO:0009611	Response to wounding	29 (12%)	2.7	7.6 × 10^–04^	Down in malignant
GO:0007010	Cytoskeleton organization	28 (11%)	2.7	8.3 × 10^–04^	Down in malignant
GO:0031224	Intrinsic to membrane	79 (41%)	1.8	1.7 × 10^–07^	Up in malignant
GO:0005794	Golgi apparatus	37 (19%)	2.5	1.5 × 10^–05^	Up in malignant
GO:0005739	Mitochondrion	45 (23%)	1.6	0.03	Up in malignant

Proteins that had a higher abundance in malignant epithelium were enriched in transmembrane proteins (41%, *p* < 0.01), proteins located in the Golgi apparatus (19.2%, *p* < 0.01), and mitochondrial proteins (23%, *p* = 0.028). Among proteins with largest fold‐changes were pro‐migratory ECM proteins including fibronectin (fold‐change = 1.9, *p* < 0.01) and versican (fold‐change = 2.7, *p* < 0.01), intracellular proteins such as the ribonucleoprotein major vault protein (fold‐change = 2.8, *p* < 0.01) and the cytokine‐induced protein signal transducer and activator of transcription 1 (fold‐change = 2.3, *p* < 0.01); along with proteins with unknown function such as pyridoxal‐dependent decarboxylase domain containing 1 (fold‐change = 3.8, *p* < 0.01; Supporting Information Table 4).

**Figure 5:**
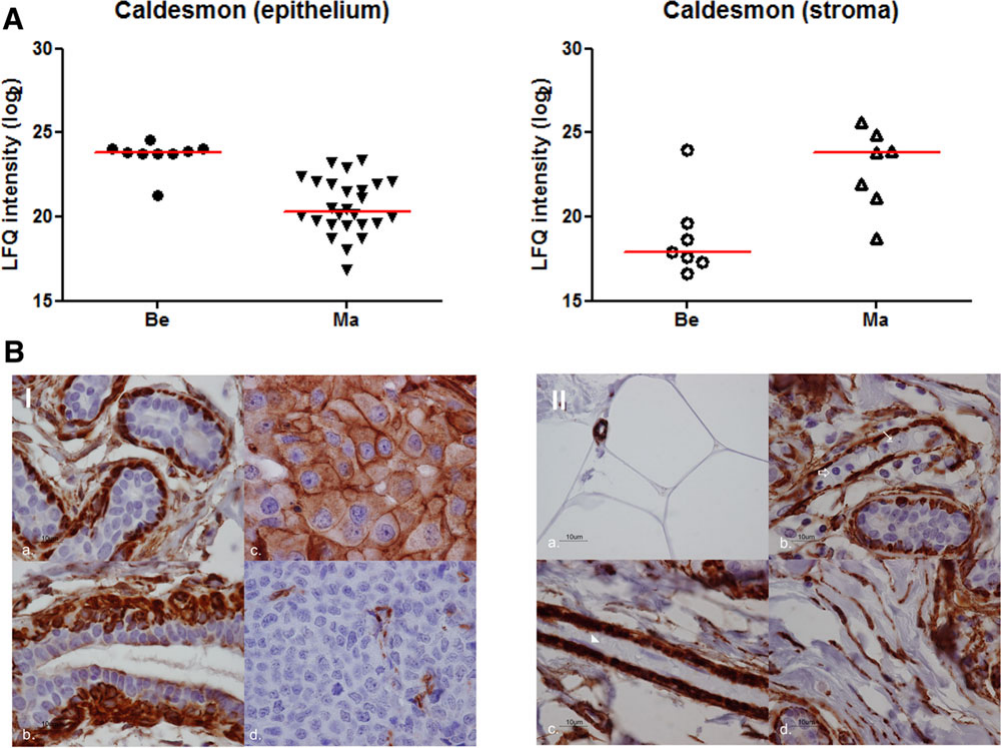
Protein abundance and immunostaining of caldesmon. (A) Protein abundance in benign and malignant epithelium and stroma. (B.I) Cytoplasm and membrane staining in; (a) myoepithelial layer of normal acini; (b) myoepithelial layer of a normal duct (some apical staining in luminal layer); (c) positive invasive breast tumor cells; (d) endothelial cells of a negative invasive breast tumor. (B.II, a) positive staining in capillaries, negative in fat cells; (b) myoepithelial layer in normal glands (negative in inflammatory cells (⇑) and tumor cells (**↑**)); (c) pericytes (negative in endothelial cells in the bloodvessel (▲); (d) positive staining in fibroblasts.

Protein abundances in microdissected stroma were more heterogeneous, most likely as a result of varying number of stromal cells in histologically malignant sections, compared to stroma from histologically normal tissue. Among extracellular proteins with largest difference in abundance were tenascin‐1 (fold‐change = 5.0, *p* = 0.03), fibronectin (fold‐change = 3.8, *p* < 0.01), and anterior gradient protein 2 (fold‐change = 3.7, *p* < 0.01; Supporting Information Table 4). Only a few markers had a significantly lower expression in malignant stroma, which included heparin cofactor 2 (fold‐change = –1.2, *p* = 0.01), hemopexin (fold‐change = –1.2, *p* = 0.02), and decorin (fold‐change = –0.9, *p* = 0.04). Approximately 25% of differentially abundant extracellular proteins between normal and malignant regions were concordant in both cells of epithelial origin and stroma. Discrepancies were mainly found for intracellular proteins, such as the cytoskeletal organizing proteins filamin‐A (fold‐change = –3.7, *p* < 0.01) in epithelium versus 2.0, *p* < 0.01) in stroma) and caldesmon (fold‐change = –4.0, *p* < 0.01 in epithelium versus 3.4, *p* < 0.01 in stroma; Fig. 5A and Supporting Information Table 4). These discrepancies may be explained by different roles of the protein in different cell types, such as inflammatory cells in stroma. To verify expression of caldesmon in different cell types, we immunohistochemically stained caldesmon in representative breast tissues (Fig. 5B). In normal ducts, caldesmon, a marker for smooth muscle differentiation, is expressed in the outer, myoepithelial, layer of cells, whereas no expression was observed in luminal cells (Fig. 5B.I). In stroma, positive staining for caldesmon was found in endothelial cells, but also in fibroblasts, reflecting a myofibroblast phenotype commonly observed in malignant stroma. Therefore, in tumor epithelium, of which the majority of samples were ER positive and of the luminal subtype, caldesmon expression is decreased, while transformation of stroma into a reactive environment likely results in a higher number of myofibroblasts and endothelial cells, and thus an overall elevated expression of caldesmon (Fig. 5B.II).

### 3.5 Origin and differences between stromal clusters

**Figure 6:**
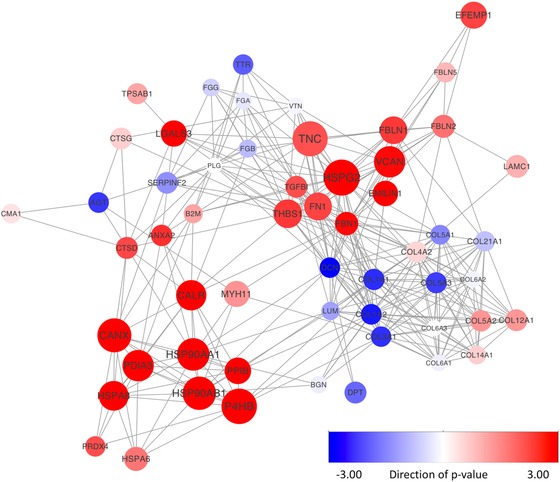
STRING protein interaction map of ECM organizing proteins detected in microdissected stroma. Selected proteins had a GO biological process annotation that included ECM organization and degradation. Node size corresponds to absolute fold change between stroma and reactive stroma, ranging from log fold change 0 to 5.5. Node color corresponds to change in abundance, with blue lower in reactive stroma and red higher. Color intensity corresponds to the significance (–log *p*‐value). Downregulated proteins were given a negative sign.

The microdissected stroma samples were separated in the PCA into two clusters, a homogeneous cluster consisting mainly of stroma dissected from benign cryosections (*n*_total_ = 10, *n*_benign_ = 6, *n*_DCIS_ = 2, and *n*_malignant_ = 2), and a more heterogeneous cluster consisting of stromal samples dissected mainly from malignant cryosections (*n*_total_ = 8, *n*_benign_ = 2, and *n*_malignant_ = 6). As was shown by the PCA (Fig. 3), the largest origin for the difference between these two groups are number and intensity of intracellular proteins, with more intracellular proteins in the “reactive” stroma group. Although proteins exclusively detected in one of the groups are likely most informative, this was heavily biased against the “reactive stroma” group, whereas no proteins were exclusively detected in the “stroma” group. Therefore, for comparative analysis between these two groups, only proteins that were detected in both groups with a minimum of five observations were included. Of the 522 selected proteins, 310 were differentially abundant between the two groups, with a very strong bias for proteins that were higher abundant in the “reactive stroma” group (*n* = 227, *p* < 0.05). The larger number of cells in the “reactive stroma” group strongly confounded results, as was evident from the higher abundance of major structural and cellular machinery proteins, such as cytoskeletal, ribosomal, and histone proteins (Supporting Information Table 8). In order to look beyond the proteins driving this bias, we further investigated proteins that had a higher abundance in the “stroma” group (*n* = 73, *p* < 0.05). Of these proteins, immunoglobulin subunits were higher in abundance, along with various stromal modeling proteins as collagens, proteoglycans, and endopeptidase inhibitors. We selected proteins that had GO biological process terms ECM organization and dissasembly, and constructed a string interaction protein network in order to highlight differences in protein abundance between the “stroma” and “reactive stroma” groups (Fig. 6). Next to the previously identified proteins that had a lower abundance in reactive stroma, such as the proteoglycans decorin and lumican, glycoproteins versican, heparin sulfate 2, and fibronectin had a higher abundance in the ‘reactive stroma’ group, as well as chaperone proteins including heat shock proteins HPS90 alpha and beta (Fig. 6). These changes, therefore, illustrate the formation of a different stromal environment accompanying malignant tumor growth.

## 4 Discussion

We have microdissected 38 breast tissues with varying stages of malignancy. Despite submicrogram amounts of starting material, nearly 3000 proteins were identified in all combined microdissected regions of interest. An advantage of microdissection is that a single region of interest can be isolated, which facilitates comparative analysis, but also reduces complexity and therefore increases proteome coverage 24. Corresponding with this, most protein identifications were made in cells of epithelial origin, which could be dissected relatively cleanly. The stromal compartment, however, was largely heterogeneously intermingled with a wide variety of cell types in varying amounts between samples. Our stromal samples could be separated into two groups, largely based on the amount of intracellular proteins. Most stromal samples from histologically malignant sections clustered in a heterogeneous group that captured most of the variance of the dataset. In contrast, stroma dissected from histologically normal tissues clustered together in a homogeneous group. Many of the additional proteins observed in the stroma of malignant sections were intracellular proteins, likely due to a transformation towards a more reactive stroma in malignant sections, characterized by an increased number of immune cells, fibroblasts and endothelial cells. This reactive stroma, or desmoplasia, has earlier been recognized and characterized, and is variable in amount and composition between tumors, independent of degree of malignancy 25.

Overall, proteomic changes between normal, benign, and malignant epithelium and stroma were readily observed, and accurately reflected common alterations in the development and progression of cancer 26. Large numbers of proteins had a lower abundance in malignant epithelium, including cell adhesion and cytoskeletal organizing proteins, reflecting the pro‐migratory phenotype of tumor cells. Furthermore, several mitochondrial proteins had a higher abundance, demonstrating increased energy metabolism. Within the stromal regions, changes mainly reflected desmoplasia, or reactivity of the stroma, as ECM proteins and cytoskeletal organizing proteins were enriched in malignant stroma as opposed to epithelium. These findings are in line with a previous study also demonstrating transition towards a more reactive stroma in malignant tissues 27, while gene expression studies have shown that the composition of this stroma is predictive for clinical outcome as well as therapy resistance 28, 29, 30. Among the differentially abundant proteins were several proteoglycans with a reduced expression in malignant stroma or epithelium, including mimecan and decorin. Proteoglycans have interesting roles in the development of cancer, as a reduced expression in stroma is associated with recurrence both in ductal carcinoma in situ and invasive carcinoma 30, 31. In contrast, higher expression of decorin is associated with chemotherapy resistance, a result of lower diffusion rates of drugs in a more dense fibrillar space 28, 32.

Disconcordant changes in expression between tissue and stroma were readily observed, such as for caldesmon, a cell differentiation marker. A loss of expression of caldesmon is associated with enhanced cellular motility due to cytoskeletal rearrangements; however, caldesmon appears to have a dual role in tumor progression, as epithelial‐to‐mesenchymal transformation results in higher expression of caldesmon in vitro 33, 34. Furthermore, an elevated level of caldesmon was associated with tamoxifen therapy resistance in recurrent ER‐positive breast cancer 35. Given that changes in expression of these and other proteins are often context dependent, microdissection clearly shows its advantage as it provides information constrained to a cell type or region of interest.

In conclusion, we showed that major phenotypical and corresponding proteomic changes were accurately captured by microdissection of different cellular regions from fresh frozen tissue sections with varying stages of disease. Protein recovery was high and unbiased, as the collected material was readily lysed and digested as is, resulting in high recovery of, for example, difficult and less soluble ECM and membrane proteins. Microdissection therefore provides a well‐suited platform for characterization of proteome changes between different clinical phenotypes. Moreover, while proteomics analysis of the bulk tumor provides a good overview, separate analysis of microdissected epithelial cells and the tumor microenvironment gives a more detailed insight into malignant transformation.

*The authors have declared no conflict of interest*.

## Supporting information

As a service to our authors and readers, this journal provides supporting information supplied by the authors. Such materials are peer reviewed and may be re‐organized for online delivery, but are not copy‐edited or typeset. Technical support issues arising from supporting information (other than missing files) should be addressed to the authors.

Table of contents

Supplemental Table 1. Overview of tissues and microdissected regions

Supplemental Table 2. Proteins uniquely identified in each microdissected region (MS2 peptide fragmentation evidence)

Supplemental table 3. Proteins corresponding to annotated clusters in figure 3

Supplemental table 4. Differentially abundant proteins between benign and malignant epithelium and benign and malignant stroma

Supplemental table 5. Enriched terms with FDR < 5% in differentially abundant proteins between benign and malignant cells of epithelial origin

Supplemental table 6. Protein identifications and missing data in microdissected regions

Supplementary table 7. Correlation matrix of modification specific peptides in anterior gradient protein 2 (O95994;AGR2) and 3 (Q8TD06;AGR3)

Supplemental table 8. Differentially abundant proteins between ‘stroma’ and ‘reactive stroma’
