# Supplementary material for: Proteomic characterization of microdissected breast tissue environment provides a protein‐level overview of malignant transformation
Source: Proteomics. 2017 Mar 7;17(5):1600213. doi: 10.1002/pmic.201600213 (PMC5347865; doi:10.1002/pmic.201600213)
Supplement: Supplementary file 1 — Table of contents [file PMIC-17-na-s001.docx]

**Supplemental information for:**

**Proteomic characterization of microdissected breast tissue environment provides a protein-level overview of malignant transformation**

René B.H. Braakman^1,5,^ *, Christoph Stingl^4^, Madeleine M.A. Tilanus-Linthorst^2^, Carolien H.M. van Deurzen^3^, Mieke A.M. Timmermans^1^, Marcel Smid^1^, John A. Foekens^1,5^, Theo M. Luider^4^, John W.M. Martens^1,5^and Arzu Umar^1,5^.

1. Department of Medical Oncology, Erasmus MC Cancer Institute, Erasmus University Medical Center, Rotterdam, The Netherlands
2. Department of Surgery, Erasmus University Medical Center, Rotterdam, The Netherlands
3. Department of Pathology, Erasmus University Medical Center, Rotterdam, The Netherlands
4. Department of Neurology, Erasmus University Medical Center, Rotterdam, The Netherlands
5. Postgraduate School of Molecular Medicine, Rotterdam, The Netherlands

* Current address: Triskelion, Zeist, The Netherlands

**Table of contents**

Figure S1: Representative overview of fresh frozen breast tissue sections used for microdissection

Figure S2: Venn diagrams of proteins identified with at least two peptides in each microdissected region

Figure S3: Distribution of PSM abundances and protein abundances in benign and malignant epithelium, and benign and malignant stroma

Figure S4: Abundance of ER, PR and HER2 compared to immunohistological results

Figure S5: Heatmap of PSMs matching to anterior gradient protein 2 (AGR2) and anterior gradient protein 3 (AGR3)

Supplemental text S1: Transfer of identifications between samples improves sequence coverage


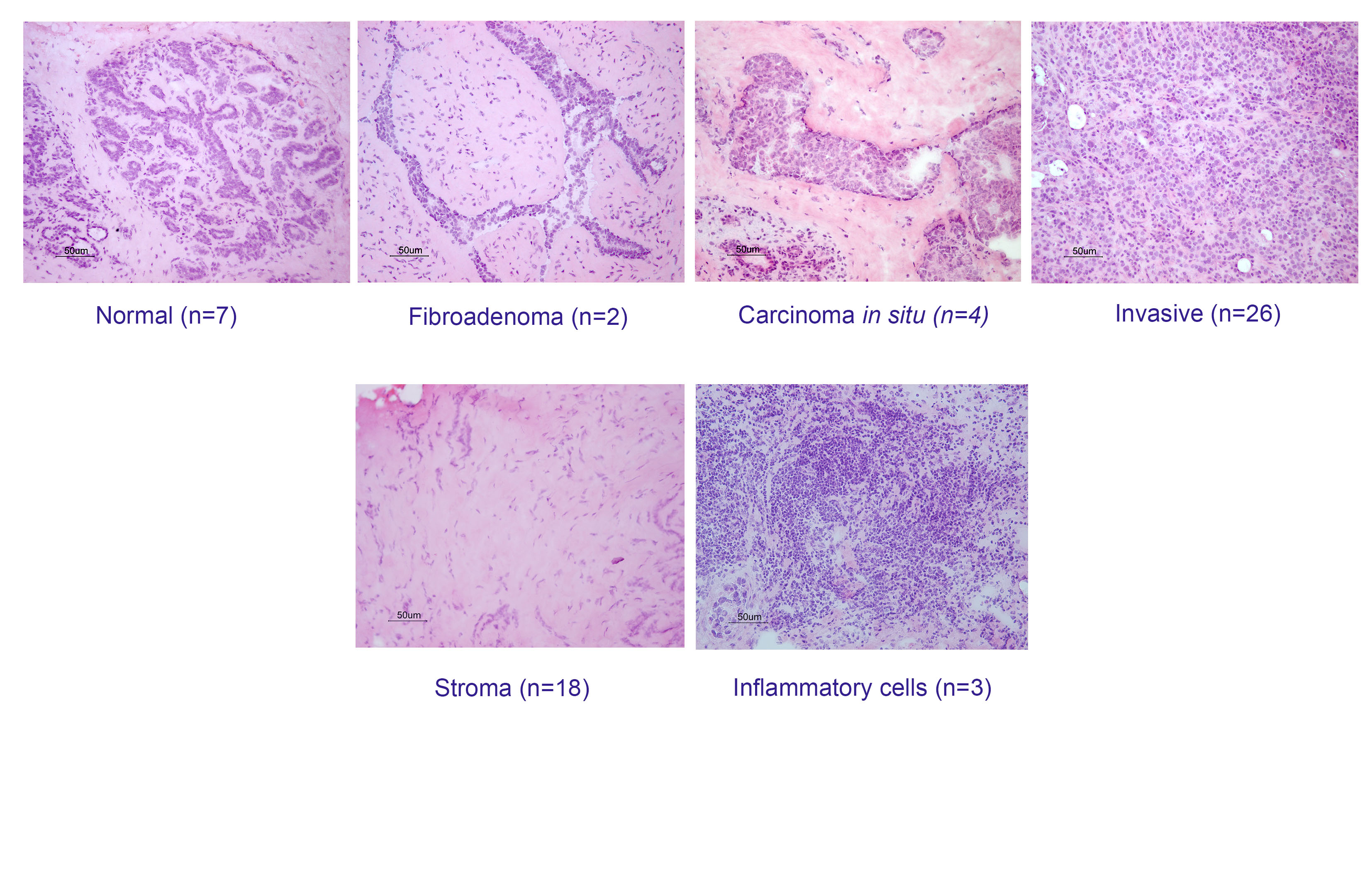


**Figure S1.** Representative overview of fresh frozen breast tissue sections used for microdissection


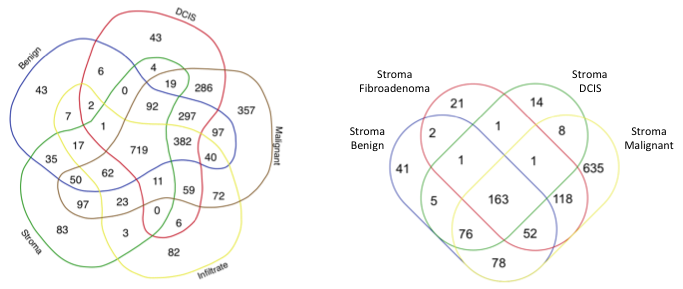


**Figure S2.** Venn diagrams of proteins identified with at least two peptides in each microdissected region.


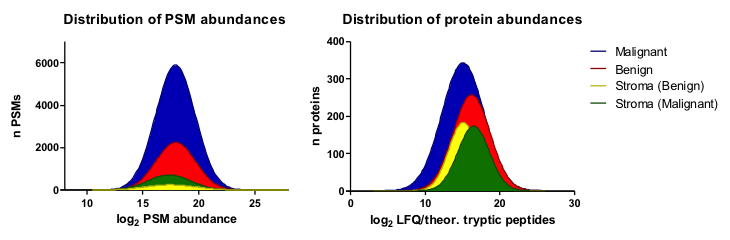


**Figure S3.** Distribution of A. PSM abundances and B. protein abundances in benign and malignant epithelium, and benign and malignant stroma.


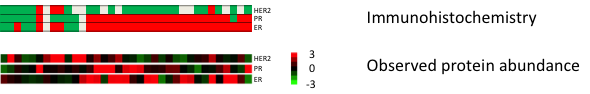


**Figure S4.** Abundance of ER, PR and HER2 (bottom heatmap) compared to immunohistological results. Red indicates a positive score in IHC results and a higher than median protein abundance across samples for each protein in the heatmap.


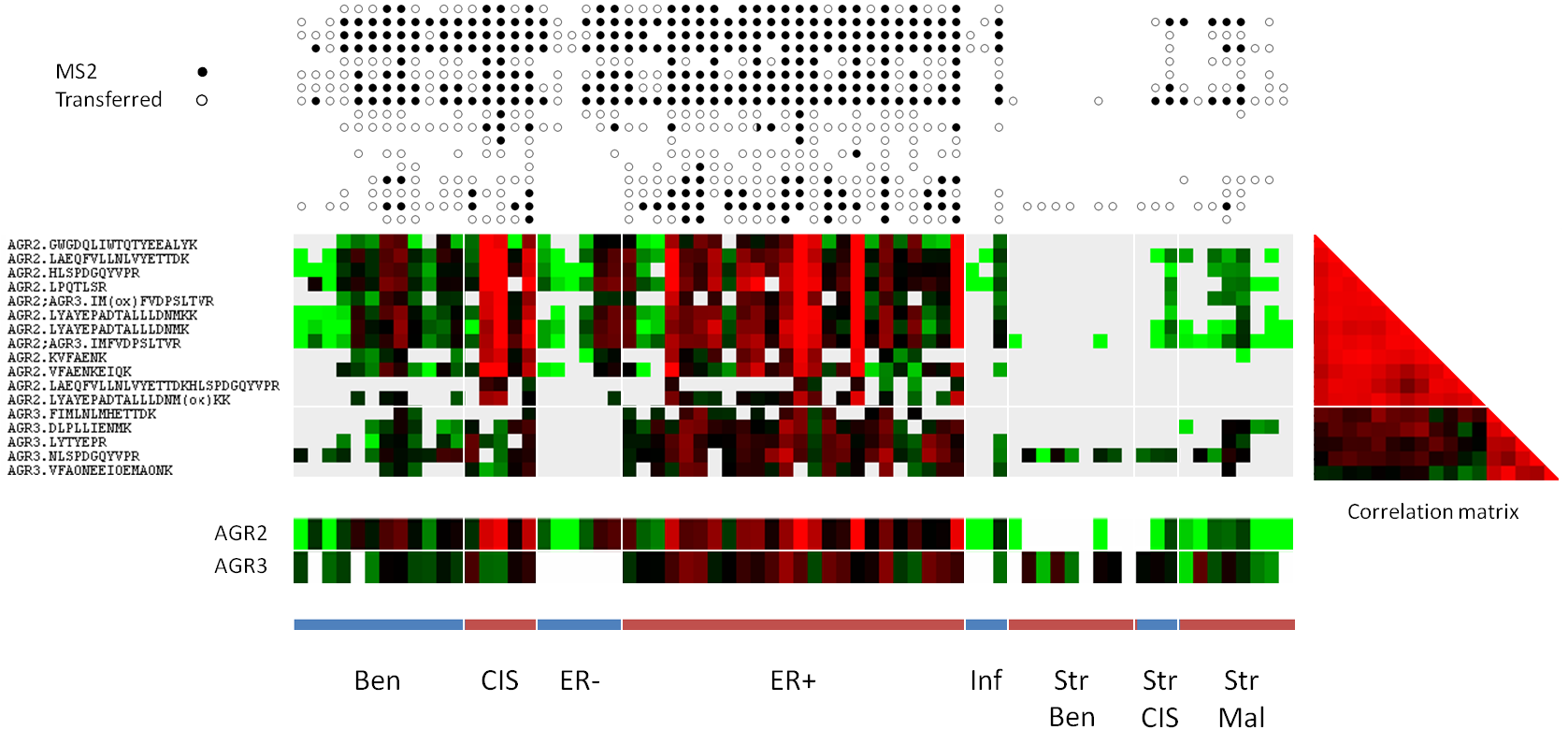


**Figure S5.** Heatmap of PSMs matching to anterior gradient protein 2 (AGR2) and anterior gradient protein 3 (AGR3), respectively, in median centered log2 abundances (red = 3, black = 0, green = -3). Bottom rows show resulting median centered inferred protein abundances (red = 4, black = 0, green = -4). White and black circles represent evidence based on fragmentation data (MS2) or evidence based on accurate mass and time (transferred), corresponding to the heatmap of PSMs. The correlation matrix shows the correlation of PSMs against each other (correlation heatmap range: red: R = 1, black: R = 0, green: R = -1). The correlation of abundances of each assigned PSM is high for both proteins (median correlation for AGR2=0.9, and median correlation for AGR3 = 0.8), irrespective of an identification based on fragmentation data or accurate mass and time data. Correlation between each protein is low (R = 0.2), ruling out spurious correlation.

**Supplemental text S1**

*Transfer of identifications between samples improves sequence coverage*

To increase protein sequence coverage in each sample, MaxQuant software allows assignments of peptides to MS1 ion traces, or ‘features’, for which fragmentation data is not present or of insufficient quality, based on retention time and accurate mass information of peptides identified in other samples. We explored the gain in identifications that could be made based on accurate mass and retention time matching, by comparing the number of protein identifications, as well as the amount of missing data with and without accurate mass and retention time matching (supplemental table 6). To avoid spurious assignments, we only considered protein groups that were identified with at least two peptides. Transferring identifications had a considerable effect on the number of proteins identified, as well as missing data. In the benign group, for example, 1,589 proteins were identified with at least two peptide observations based on fragmentation spectra. After transfer of identifications, this increased to 2,203 protein groups, even when only considering protein groups identified with two different peptides. Missing data in the benign group decreased from 67% to 28% (supplemental table 6). To determine accuracy of matching, we compared peptide abundances of AGR2 and AGR3, two proteins involved in breast cancer progression and metastasis. These proteins were identified with 10 and 5 non-degenerate peptide sequences, respectively, as well as two overlapping sequences. Evidence for these peptides varied, e.g. AGR2 peptide LAEQFVLLNVYETTDK was identified in all malignant tumor samples with MS2 evidence, whereas the miscleaved peptide VFAENKEIQK was detected in 23 out of 24 samples, but with MS2 evidence in only 2 samples. Regardless of type of evidence, peptide abundances were significantly correlated within each protein (average R = 0.86 for AGR2 and R = 0.73 for AGR3, figure S-5 and supplemental table 7), and transferred identifications therefore appeared to accurately contribute to estimated protein abundances.
